# Stronger associations of affectivity than social leisure activities with cognitive impairment: a 10-year trajectory study of Chinese older adults

**DOI:** 10.3389/fpsyt.2026.1842578

**Published:** 2026-05-19

**Authors:** Yelin Gu, Jiaqi Zong, Yueyang Wu, Qin Wang, Lingling Pan, Jianlin Gao

**Affiliations:** 1 School of Public Health, Nantong University, Nantong, Jiangsu, China; 2 Institute for Health Development, Nantong University, Nantong, Jiangsu, China; 3 Nantong Center for Disease Control and Prevention, Nantong, Jiangsu, China; 4 Affiliated Hospital of Nantong University, Nantong, Jiangsu, China

**Keywords:** cognitive function, cohort analysis, group-based trajectory models, positive and negative affectivity, social leisure activities, successful aging

## Abstract

**Background:**

Both positive and negative affectivity (PNA) and social leisure activities (SLA) are significantly associated with cognitive function, yet their relative strength of association with long-term cognitive decline remains unclear. Exploring this issue has significant implications for designing precise and effective cognitive health promotion programs in the future.

**Methods:**

The data were derived from the Chinese Longitudinal Healthy Longevity Survey (CLHLS). Our analysis included 1,847 older adults (≥65 years) without diagnosed dementia at baseline, who were followed from 2008 to 2018. Using Group-Based Trajectory Models (GBTM), participants were classified based on their PNA and SLA trajectories to identify distinct subgroups. Binomial logistic regression analyzed associations between these groups and cognitive function, with Likelihood Ratio and Wald Tests comparing their relative strengths of association.

**Results:**

The “High-rapid-increasing PNA” group exhibited a 264% higher risk of cognitive impairment compared to the “Low-stable-increasing PNA” group (OR = 3.64, 95% CI: 2.92, 4.52). The “Low-stable SLA” group was associated with increased cognitive impairment risk (OR = 1.66, 95% CI: 1.32, 2.08). PNA demonstrated a stronger association with cognitive function than SLA [Likelihood Ratio Test: Δχ²(1) = 137.37, p < 0.001]. However, a formal test for multiplicative interaction was not statistically significant (OR = 0.88, 95% CI: 0.57, 1.35).

**Conclusion:**

PNA demonstrated a stronger association with cognitive function in older adults than SLA. Affective factors may be a critical, and potentially underutilized, target for cognitive health interventions in aging populations.

## 1 Introduction

Population aging is an urgent global public health issue that presents particularly severe challenges in China, placing sustained pressure on socioeconomic and healthcare systems (1). According to data from the World Health Organization (WHO), over 55 million people worldwide currently suffer from dementia, with nearly 10 million new cases diagnosed each year. A national cross-sectional survey in China revealed a crude prevalence rate of 9.1% among individuals aged 65 and older (2). Research indicates that cognitive decline in older adults significantly increases the risk of developing dementia or mild cognitive impairment (3). Because interventions targeting cognitive decline can be effective, preventing or slowing cognitive decline in older adults not only enhances their quality of life and that of their caregivers but also helps reduce healthcare and financial burdens (4).

Positive affective characteristics manifest as high energy and pleasurable engagement, reflecting an individual’s enthusiasm, liveliness, and acute alertness (5, 6). In contrast, negative affectivity constitutes a broad spectrum of subjective distress and unpleasant experiences, encompassing various aversive affective states such as anger, fear, and tension (7). Previous research has suggested that certain affective states, such as anxiety and loneliness, are associated with accelerated cognitive aging. Anxiety is a recognized risk factor for cognitive decline and dementia in community-based populations (8). Moreover, a meta-analysis found that 6.5% of the overall risk of cognitive impairment could be attributed to anxiety, while 7.9% of the risk of dementia could be attributed to anxiety (9). A population-based cohort study conducted in the United States indicates that prolonged loneliness from middle age through later life may significantly accelerate memory decline (10). However, existing evidence has largely examined specific affective dimensions in isolation and thus lacks an assessment of the combined effects of positive and negative affectivity (PNA) on cognitive decline.

Social participation refers to the extent to which individuals engage in a variety of social roles and relationships (11). Leisure activities refer to pursuits individuals engage in outside of work or daily responsibilities for the purpose of enjoying or enhancing well-being (12). Numerous studies have linked participation in social leisure activities (SLA) to better physical and mental health, slower cognitive decline, and lower dementia risk (13–15). Among Asian populations, culturally distinctive activities such as mahjong and Chinese chess engage multiple cognitive domains including attention, memory and calculation skills, providing not only intellectual stimulation but also interpersonal interaction and social connections (16). To overcome the limitations of previous studies that employed simplistic “yes/no” responses or frequency measures, this study categorized social activities into seven dimensions: gardening, outdoor pursuits, pet/poultry ownership, reading, board and card games, audiovisual entertainment, and organized social activities.

Although both affective and activity factors have been demonstrated to exert significant effects on cognitive health, a core question remains unresolved: which of the two demonstrates a stronger statistical association with cognitive impairment? Socio-emotional selectivity theory suggests that as older adults perceive their remaining time as limited, they prioritize emotionally meaningful goals over those offering future benefits (17). This theory explains their preferences in target selection, but does not confirm whether such preferences equate to superior health benefits. For older adults with limited time and energy, which shows a stronger link to cognitive health: optimizing affective well-being or encouraging social leisure activities? Moreover, beyond this simple comparison, it is unknown whether PNA and SLA operate as independent protective factors or interact, such that their combined presence or absence is associated with a risk profile that is greater or lesser than the sum of its parts. Current evidence, by examining these factors in isolation, fails to provide a definitive answer.

Therefore, based on the nationally representative China Longitudinal Healthy Longevity Survey (CLHLS), we aim to: 1) investigate the respective associations of PNA and SLA with cognitive decline; 2) compare the magnitude of their associations to determine which factor demonstrates a stronger association; and 3) examine their combined effects by examining how specific combinations of PNA and SLA trajectories are associated with differential risk levels.

## 2 Materials and methods

### 2.1 Study data and participants

This study utilized data from the China Longitudinal Healthy Longevity Survey (CLHLS), a prospective cohort focusing on adults aged ≥65 years. Employing a multistage cluster sampling method, the CLHLS covers 23 out of China’s 31 provinces, encompassing roughly 85% of the country’s population (18, 19). The cohort design incorporates follow-up assessments every 3 to 4 years. All enrolled participants gave their written informed consent to participate in both the initial and subsequent surveys. This study has been approved by the Biomedical Ethics Committee of Peking University, Beijing, China (IRB00001052-13074).

This study used data from the fifth wave (2008) as the baseline, with data from the sixth wave (2011), seventh wave (2014), and eighth wave (2018) serving as follow-up tracking. Participants were excluded if they were aged < 65 years, had missing values in the PNA, SLA, or covariates, or had been diagnosed with dementia at baseline. The final analysis included 1,847 participants, as detailed in the sample selection flowchart (**Supplementary Figure 1**).

### 2.2 Cognitive function assessment

Researchers used the Chinese version of the Mini-Mental State Examination (MMSE) to assess participants’ cognitive function. This scale, adapted from the original version by Folstein, is a widely recognized tool for evaluating cognitive status in older adults (20). The Chinese MMSE covers five cognitive domains (orientation, memory, calculation, recall, and language) through its 23 items. This version was specifically adapted for Chinese older adults. To enhance cultural and socioeconomic relevance, certain content has been modified or removed. For example, participants were asked to orally list as many food names as possible as an alternative to constructing complete sentences. The math problems were simplified, with one time-orientation question and four location questions removed (21). The MMSE employs a binary scoring system, where each correct response is scored as 1 point and each incorrect response as 0 points. The total score is calculated by combining all items, with higher scores indicating better cognitive function. A total score below 18 points is considered an indicator of cognitive impairment (22, 23). This relatively low cutoff was adopted because the study population had very limited formal education, with a median of only 1 year of schooling and nearly half of participants having received no formal education.

### 2.3 Positive and negative affectivity assessment

Positive and negative affectivity were assessed using a dual-dimensional scale. The seven items included in the CLHLS were conceptually informed by the Positive and Negative Affect Schedule (PANAS) framework (7). Rather than directly translating the PANAS, CLHLS researchers designed culturally adapted items specifically tailored to the Chinese older adult population, paying particular attention to Confucian cultural values and intergenerational relationship dynamics prevalent in Chinese society (24). Positive affectivity included four subdimensions: philosophical optimism, autonomy, sense of youthfulness and clean preference. Negative affectivity comprised three subdimensions: tension/fear, loneliness, and sense of worthlessness. All items were rated on a 5-point scale (1 = always, 5 = never). Total scores spanned 7 to 35, and elevated scores corresponded to more intense negative affective states. In the present sample, the scale demonstrated acceptable internal consistency (Cronbach’s α = 0.828). The complete question set and scores are available in **Supplementary Table 1**.

### 2.4 Social leisure activities index

The Social Leisure Activities Index was derived from seven items in the CLHLS questionnaire that assess participation in common leisure and social activities among Chinese older adults. The index comprises the following seven categories: gardening; outdoor pursuits (such as square dancing and tai chi); pet/poultry ownership; reading; board and card games (such as playing cards and mahjong); audiovisual entertainment (such as listening to or watching radio and television); and organized social activities. While some activities are inherently social (e.g., organized social activities, board and card games), others may be pursued more solitarily but still within the broader context of leisure engagement (e.g., reading, gardening). Collectively, these items encompass the major forms of leisure and social engagement common among Chinese older adults, and are consistent with the established understanding that leisure activities can foster a sense of achievement and self-fulfillment, stimulate creativity and service to others, provide opportunities for relaxation and personal growth, and help older adults alleviate emptiness and boredom (25, 26). A composite index (range 0-7) was calculated by summing the number of activities participated in, with each activity coded as either 0 (no) or 1 (yes).

### 2.5 Covariates

The study collected data on demographic characteristics, health conditions, and lifestyle factors through a questionnaire. These encompassed: age; gender; geographic residence; years of education (continuous) and illiteracy status (yes/no). Health status was categorized into four groups based on BMI values (normal weight, underweight, overweight, or obesity). Lifestyle factors considered in the study included smoking and alcohol consumption. For each factor, participants were categorized into three groups (never, former, current).

### 2.6 Statistical analysis

Group-Based Trajectory Models (GBTM) were used to identify distinct developmental patterns of PNA and SLA across four time points (2008, 2011, 2014, 2018). GBTM is a finite mixture modeling approach that assumes the population is composed of latent subgroups, each following a distinct trajectory over time (27). For each outcome, we specified a censored normal distribution to account for the bounded and approximately continuous nature of the measures. Trajectories were modeled as polynomial functions of time. Models with one to four groups were sequentially fitted; five-group models failed to converge and were excluded. Solutions that failed to extract the specified number of distinct trajectory groups (i.e., partially converged) were also excluded from model comparisons. Model selection was based on the Bayesian Information Criterion (BIC), supplemented by average posterior probabilities (>0.7 for each group) and interpretability of the resulting trajectories. For PNA, the two-class solution was selected as optimal (BIC = 41,979.62; see **Supplementary Table 2**). For SLA, the two-class solution was retained based on the lowest BIC among converged models (BIC = 25,528.44; see **Supplementary Table 3**). Accordingly, a two-trajectory model was used for subsequent analyses.

Data from all four waves were pooled for analysis. Logistic regression models were fitted to examine the associations of PNA and SLA with cognitive impairment assessed at each wave. Odds ratios (ORs) and 95% confidence intervals (CIs) were calculated. Models 1 and 2 examined the association of PNA with cognitive impairment, unadjusted and adjusted for sociodemographic characteristics, respectively. Models 3 and 4 examined the association of SLA with cognitive impairment, unadjusted and adjusted for sociodemographic characteristics, respectively. Model 5 included both PNA and SLA simultaneously to assess their independent contributions to cognitive impairment. To formally test for a multiplicative interaction between PNA and SLA, a product term (High-rapid-increasing PNA × Low-stable SLA) was included in the fully adjusted model.

The results of Models 2, 4, and 5 were visualized using forest plots. Likelihood ratio tests were conducted to compare the goodness of fit between models and assess the contributions of PNA and SLA. The Wald test in Model 5 compared the regression coefficients of PNA and SLA to assess whether there is a significant difference in their effects on cognitive function. In additional analyses, participants were classified by their distinct PNA and SLA trajectories into four groups to further examine the combined association with cognitive function. Additionally, generalized estimating equation (GEE) models were fitted with MMSE scores treated as a continuous outcome across all four survey waves, using an exchangeable working correlation structure to account for within-subject correlation. Sensitivity analyses were conducted to address potential sources of bias and ensure the reliability of our results. First, the associations of PNA and SLA trajectories with cognitive impairment were examined cross-sectionally using only the 2008 baseline data to assess whether the observed longitudinal associations were evident cross-sectionally. Moreover, this study employed education-specific cutoff points to stratify MMSE scores: 18 points for those with no formal education, 20 points for elementary education (1–6 years), and 24 points for secondary education and above (>6 years) (28).

## 3 Results

### 3.1 Estimated PNA trajectory modeling

**Figure 1:**
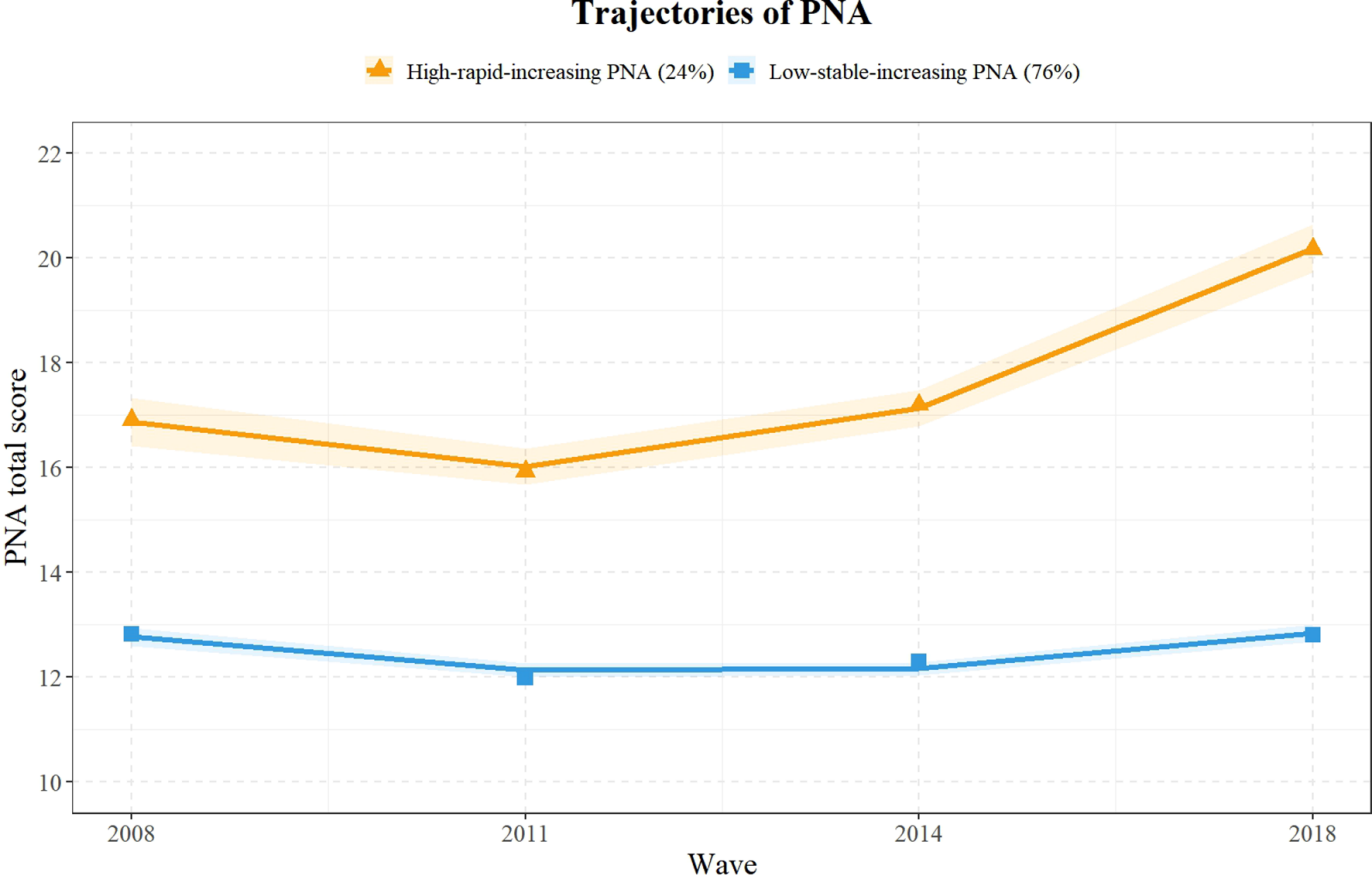
Trajectories of positive and negative affectivity (PNA).

**Figure 1** illustrates the trajectories of two primary PNA classes. Category 1, labeled as “High-rapid-increasing PNA,” comprised 449 participants (24.31%), whereas Category 2, termed “Low-stable-increasing PNA,” included 1,398 participants (75.69%). The figure presents the GBTM-estimated trajectories (solid lines), observed mean scores at each wave (triangles for High-rapid-increasing PNA, squares for Low-stable-increasing PNA), and 95% confidence bands (shaded areas). These classifications highlight distinct patterns of PNA changes over the study period, clearly showing how PNA levels changed across participant groups. The corresponding fitting statistics for the PNA trajectories are provided in **Supplementary Table 2**.

### 3.2 Estimated SLA trajectory modeling

**Figure 2:**
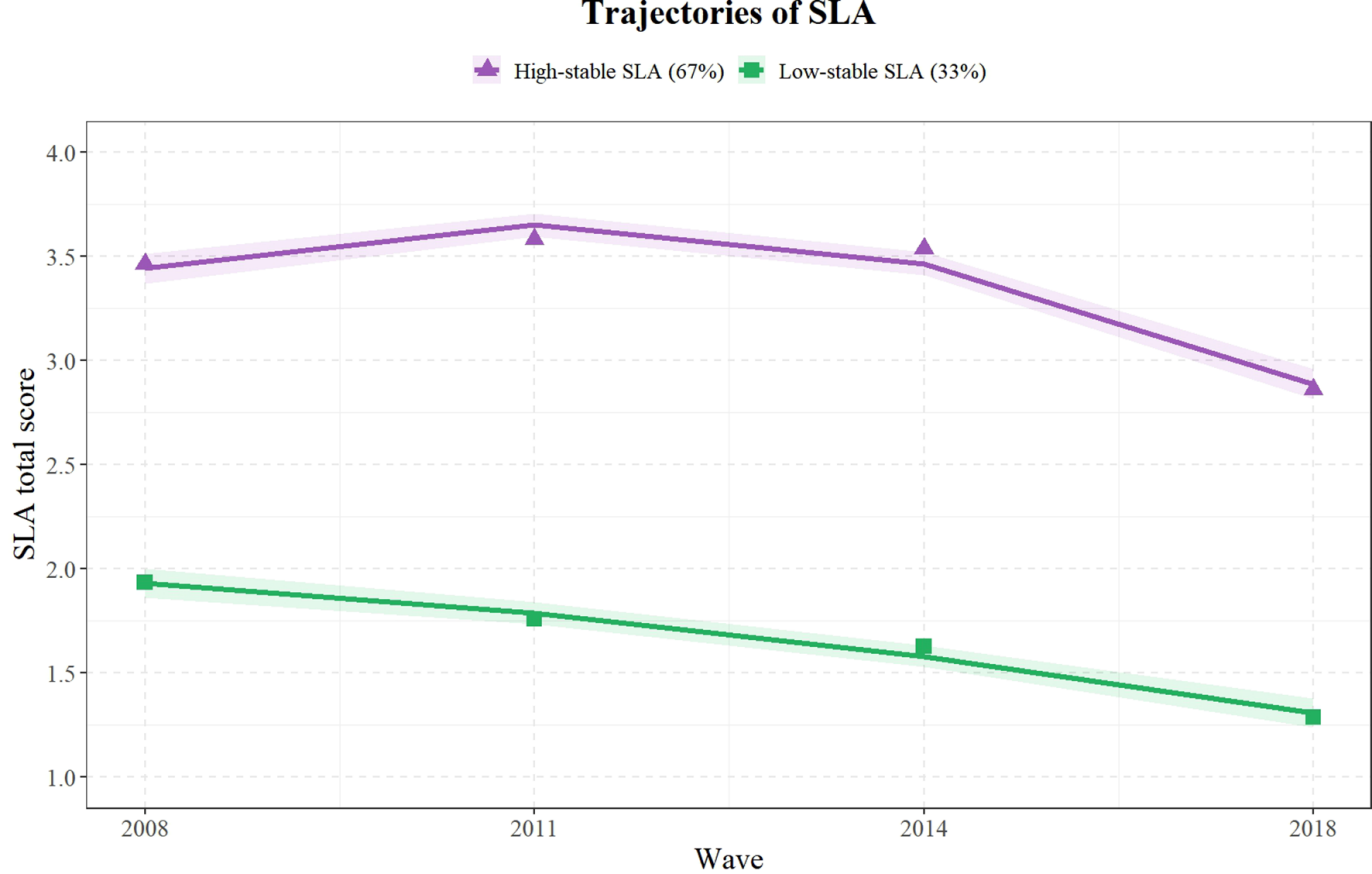
Trajectories of social leisure activities (SLA).

**Figure 2** illustrates two trajectory patterns of SLA. Trajectory 1 represents the “High-stable SLA,” accounting for 66.97% of the total study population. Trajectory 2 corresponds to “Low-stable SLA” and accounts for 33.03% of the total study population. The figure displays the GBTM-estimated trajectories (solid lines), observed mean scores at each wave (colored triangles and squares), and 95% confidence bands (shaded areas). These patterns reveal distinct stable SLA trajectories among participants, highlighting differences in SLA levels over time. Fitting statistics for SLA trajectories are in **Supplementary Table 3**.

### 3.3 Baseline characteristics of participants based on PNA and SLA

The baseline characteristics of the 1,847 participants who completed the 10-year follow-up are summarized in **Table 1**. In brief, 51.60% were male, the median age at baseline was 73 years, 67.46% resided in rural areas, and 52.68% had formal education, with a mean MMSE score of 26.90. Descriptive characteristics of participants at each follow-up wave are presented in **Supplementary Table 4**. Longitudinal changes in MMSE scores from 2008 to 2018, stratified by PNA and SLA trajectory groups, are shown in **Supplementary Table 5**.

**Table 1:** Baseline characteristics of participants by types of PNA and SLA.

Variables	Overall n=1847	Low-stable-increasing PNA n=1398	High-rapid-increasing PNA n=449	p-Value	High-stable SLA n=1237	Low-stable SLA n=610	p-Value
MMSE score*	26.90 ± 4.55	27.55 ± 3.30	24.88 ± 6.79	<0.001	27.71 ± 3.48	25.25 ± 5.84	<0.001
Age, M (P25, P75)	73 (69, 80)	72 (68, 78)	78 (71, 84)	<0.001	72 (68, 78)	77 (71, 83)	<0.001
Years of education, M(P25, P75)	1 (0, 5)	2 (0, 6)	0 (0, 2)	<0.001	3 (0, 6)	0 (0, 2)	<0001
Cognitive function							
Normal cognitive function	1774 (96.05)	1369(77.17)	405(22.83)	<0.001	1212 (68.32)	562 (31.68)	<0.001
Cognitive impairment	73 (3.95)	29 (39.73)	44(60.27)		25 (34.25)	48 (65.75)	
Sex, n (%)							
Male	894 (48.40)	752 (84.12)	142(15.88)	<0.001	712 (79.64)	182 (20.36)	<0.001
Female	953 (51.60)	646 (67.79)	307(32.21)		525 (55.09)	428 (44.91)	
Residency, n (%)							
City	241 (13.05)	194 (80.50)	47 (19.50)	0.007	205 (85.06)	36 (14.94)	<0001
Urban	360 (19.49)	288 (80.00)	72 (20.00)		250 (69.44)	110 (30.56)	
Rural	1246 (67.46)	916 (73.52)	330 (26.48)		782 (62.76)	464 (37.24)	
Illiteracy, n (%)							
No	973 (52.68)	820 (84.28)	153 (15.72)	<0.001	777 (79.86)	196 (20.14)	<0.001
Yes	874 (47.32)	578 (66.13)	296 (33.87)		460 (52.63)	414 (47.37)	
BMI, n (%)							
Normal weight	1031 (55.82)	814 (78.95)	217 (21.05)	<0.001	716 (69.45)	315 (30.55)	0.001
Underweight	694 (37.57)	484 (69.74)	210 (30.26)		428 (61.67)	266 (38.33)	
Overweight	44 (2.38)	37 (84.09)	7 (15.91)		34 (77.27)	10 (22.73)	
Obesity	78 (4.22)	63 (80.77)	15 (19.23)		59 (75.64)	19 (24.36)	
Smoking status, n (%)							
Never smoker	1169 (63.29)	824 (70.49)	345 (29.51)	<0.001	699 (59.79)	470 (40.21)	<0.001
Former smoker	422 (22.85)	357 (84.60)	65 (15.40)		340 (80.57)	82 (19.43)	
Current smoker	256 (13.86)	217 (84.77)	39 (15.23)		198 (77.34)	58 (22.66)	
Alcohol consumption, n (%)							
Never drinker	1189 (64.37)	857 (72.08)	332 (27.92)	<0.001	739 (62.15)	450 (37.85)	<0.001
Former drinker	236 (12.78)	183 (77.54)	53 (22.46)		166 (70.34)	70 (29.66)	
Current drinker	422 (22.85)	358 (84.83)	64 (15.17)		332 (78.67)	90 (21.33)	

Numbers (%) were reported.

*mean (standard deviation) was reported.

PNA, Positive and negative affectivity; SLA, Social leisure activities.

### 3.4 Association of PNA and SLA with cognitive function

**Table 2**, **Figure 3** present the odds ratios (ORs) for High-rapid-increasing PNA compared to Low-stable-increasing PNA and Low-stable SLA compared to High-stable SLA. In the PNA crude model, High-rapid-increasing PNA was associated with a six-fold higher odds of cognitive impairment (OR = 6.54, 95% CI: 5.37, 7.96). In the SLA crude model, Low-stable SLA was associated with a four-fold higher odds of cognitive impairment (OR = 4.06, 95% CI: 3.34, 4.94). After adjusting for sociodemographic characteristics, both associations remained statistically significant, with attenuated but substantial effect sizes (PNA adjusted model: OR = 4.08, 95% CI: 3.30, 5.05; SLA adjusted model: OR = 2.26, 95% CI: 1.82, 2.81).

**Table 2:** Logistic regression models for PNA, SLA and cognitive function.

Models	Variables	Normal cognitive function	Cognitive impairment
PNA crude model OR (95% CI)	High-rapid-increasing PNA (Ref.Low-stable-increasing PNA)	Reference	6.54 (5.37, 7.96)
PNA adjusted model OR (95% CI)	High-rapid-increasing PNA (Ref. Low-stable-increasing PNA)	Reference	4.08 (3.30, 5.05)
SLA crude model OR (95% CI)	Low-stable SLA (Ref. High-stable SLA)	Reference	4.06 (3.34, 4.94)
SLA adjusted model OR (95% CI)	Low-stable SLA (Ref. High-stable SLA)	Reference	2.26 (1.82, 2.81)
PNA and SLA simultaneous model OR (95% CI)	High-rapid-increasing PNA (Ref. Low-stable-increasing PNA)	Reference	3.64 (2.92, 4.52)
	Low-stable SLA (Ref. High-stable SLA)	Reference	1.66 (1.32, 2.08)
PNA and SLA interaction model OR (95% CI)	High-rapid-increasing PNA (Ref. Low-stable-increasing PNA)	Reference	3.92 (2.82, 5.44)
	Low-stable SLA (Ref. High-stable SLA)	Reference	1.78 (1.29, 2.46)
	High-rapid-increasing PNA ×Low-stable SLA (Ref. Low-stable-increasing PNA × High-stable SLA)	Reference	0.88 (0.57, 1.35)

OR, Odds ratio.

Adjusted models were were adjusted for age (continous), sex (male or female), residency (city/urban/rural), illiteracy (yes/no), BMI (normal weight, underweight, overweight or obesity), smoking and drinking status (never, former or current smokers/drinkers).

PNA, Positive and negative affectivity; SLA, Social leisure activities.

**Figure 3:**
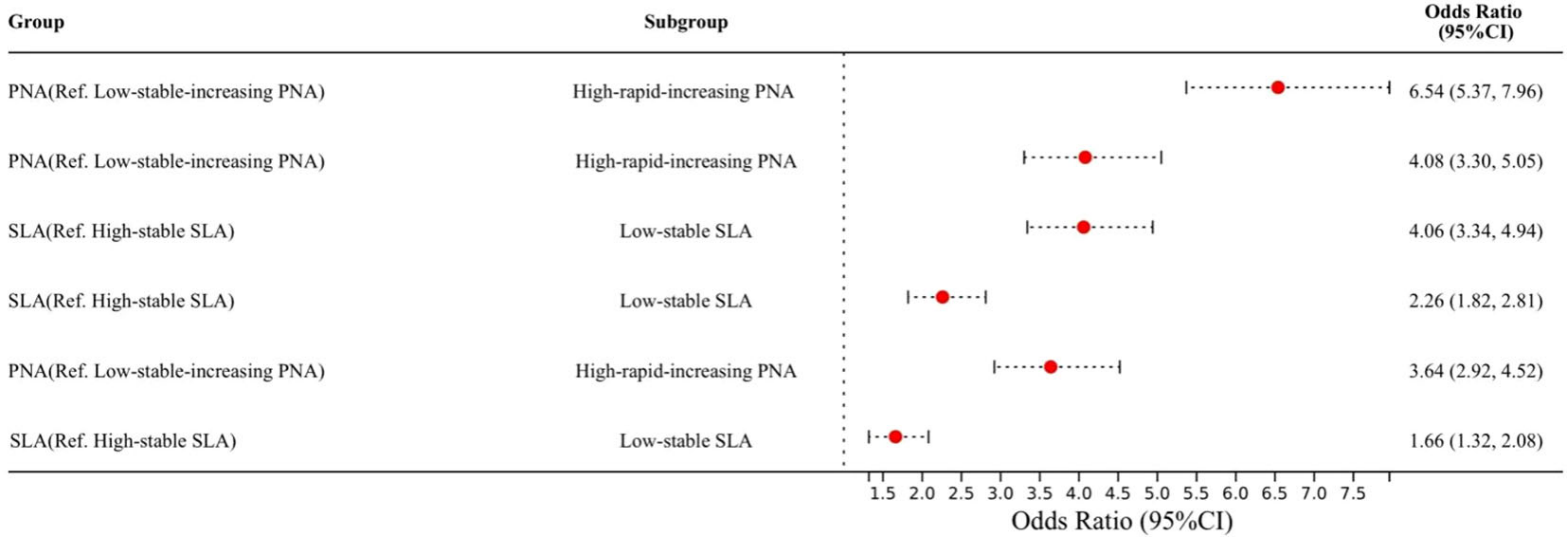
Forest plot showing odds ratios (OR) of cognitive impairment for trajectories of PNA and SLA.

To assess the independent contributions of PNA and SLA, a simultaneous model including both trajectories was fit. In this model, High-rapid-increasing PNA (OR = 3.64, 95% CI: 2.92, 4.52) and Low-stable SLA (OR = 1.66, 95% CI: 1.32, 2.08) were both independently associated with cognitive impairment, with the point estimate for PNA being larger in magnitude. The primary analysis results were consistent across the following sensitivity analyses: cross-sectional analysis using 2008 baseline data (**Supplementary Table 6**), and using education-specific MMSE cutoff points (**Supplementary Table 7**).

A multiplicative interaction between PNA and SLA trajectories was further formally tested by including a product term (High-rapid-increasing PNA × Low-stable SLA) in the fully adjusted model. The interaction term was not statistically significant (OR = 0.88, 95% CI: 0.57, 1.35), suggesting no multiplicative interaction between PNA and SLA trajectories.

### 3.5 Likelihood ratio test and Wald test

The likelihood ratio test in **Table 3** indicated that PNA significantly improved model fit when added to a model containing only SLA and covariates (Δχ²(1) = 137.37, p < 0.001). Conversely, excluding SLA from Model 5 resulted in a smaller but still significant reduction in fit (Δχ²(1) = 19.07, p < 0.001). These results suggest that both variables are independently associated with cognitive function, but PNA demonstrates a stronger association. The association between PNA and cognitive impairment is significantly stronger than that of SLA (Wald test for coefficient equality: χ²(1) = 19.48, p < 0.001).

**Table 3:** Comparison of model fit by using likelihood ratio test.

Model Comparison	Δdf	Δχ²	p-value	ΔAIC	ΔBIC
PNA and SLA simultaneous model vs. SLA adjusted model	1	137.37	<0.001	-135.01	-128.47
PNA and SLA simultaneous model vs. PNA adjusted model	1	19.07	<0.001	-17.08	-10.17

Δ AIC/BIC: Difference in Akaike/Bayesian Information Criterion between full and reduced models.

PNA, Positive and negative affectivity; SLA, Social leisure activities.

### 3.6 Additional analysis

1056 participants (57.17%) had Low-stable-increasing PNA and High-stable SLA, 181 participants (9.80%) had High-rapid-increasing PNA and High-stable SLA, 342 participants (18.52%) had Low-stable-increasing PNA and Low-stable SLA, and 268 participants (14.51%) had High-rapid-increasing PNA and Low-stable SLA. Additional analysis results (**Table 4**) showed that, compared to Low-stable-increasing PNA and High-stable SLA, High-rapid-increasing PNA and High-stable SLA (OR = 3.92, 95% CI: 2.82, 5.44), Low-stable-increasing PNA and Low-stable SLA (OR = 1.78, 95% CI: 1.29, 2.46), and High-rapid-increasing PNA and Low-stable SLA (OR = 6.10, 95% CI: 4.59, 8.11) were all associated with significantly higher odds of cognitive impairment.

**Table 4:** Associations of PNA and SLA with cognitive impairment.

Characterize	Normal cognitive function	Cognitive impairment OR (95% CI)
Ref: Low-stable-increasing PNA and High-stable SLA		
High-rapid-increasing PNA and High-stable SLA	Reference	3.92 (2.82, 5.44)
Low-stable-increasing PNA and Low-stable SLA	Reference	1.78 (1.29, 2.46)
High-rapid-increasing PNA and Low-stable SLA	Reference	6.10 (4.59, 8.11)

OR, Odds ratio.

PNA, Positive and negative affectivity; SLA, Social leisure activities.

Generalized estimating equation (GEE) models were fitted with MMSE scores treated as a continuous outcome across the four survey waves (2008, 2011, 2014, and 2018). High-rapid-increasing PNA was associated with lower MMSE scores (adjusted β = -2.71, 95% CI: -2.98, -2.44), as was Low-stable SLA (adjusted β = -1.60, 95% CI: -1.85, -1.34). In the simultaneous model including both trajectories, the association with MMSE scores was larger in magnitude for PNA (adjusted β = -2.45, 95% CI: -2.72, -2.18) than for SLA (adjusted β = -1.07, 95% CI: -1.33, -0.82) (**Table 5**).

**Table 5:** The associations between PNA, SLA and cognitive function, using the generalized estimating equation model.

	Variable	Normal cognitive function	Cognitive impairment
PNA crude model β (95% CI)	High-rapid-increasing PNA (Ref. Low-stable-increasing PNA)	Reference	-4.01 (-4.28, -3.73)
PNA adjusted model β (95% CI)	High-rapid-increasing PNA (Ref. Low-stable-increasing PNA)	Reference	-2.71 (-2.98, -2.44)
SLA crude model β (95% CI)	Low-stable SLA (Ref. High-stable SLA)	Reference	-2.99 (-3.24, -2.74)
SLA adjusted model β (95% CI)	Low-stable SLA (Ref. High-stable SLA)	Reference	-1.60 (-1.85, -1.34)
PNA and SLA simultaneous model β (95% CI)	High-rapid-increasing PNA (Ref. Low-stable-increasing PNA)	Reference	-2.45 (-2.72, -2.18)
	Low-stable SLA (Ref. High-stable SLA)	Reference	-1.07 (-1.33, -0.82)

OR, Odds ratio. Adjusted models were adjusted for age (continous), sex (male or female), residency (city/urban/rural), illiteracy (yes/no) , BMI (normal weight, underweight, overweight or obesity), smoking and drinking status (never/ former/current smokers/drinkers). PNA, Positive and negative affectivity; SLA, Social leisure activities.

## 4 Discussion

This study, based on data from the Chinese Longitudinal Health Longevity Survey (CLHLS), tracked the trajectories of positive and negative affectivity (PNA) and social leisure activities (SLA) among 1,847 nationally representative Chinese older adults across four waves. The study also examined the associations between PNA and SLA and cognitive decline. To our knowledge, this is the first study to directly compare the magnitude of associations between these two constructs and cognitive impairment in this population. Our analysis highlights three key findings: First, High-rapid-increasing PNA was associated with substantially higher odds of cognitive impairment compared to Low-stable-increasing PNA. Second, Low-stable SLA was associated with higher odds of cognitive impairment compared to High-stable SLA. Third, in direct comparison, the association with cognitive impairment was stronger for High-rapid-increasing PNA than for Low-stable SLA.

A key finding of our study is that High-rapid-increasing PNA was associated with higher odds of cognitive impairment compared to Low-stable-increasing PNA. These findings are consistent with the broader literature suggesting that negative affectivity is adversely associated with cognitive function and provide empirical support for the continued integration of affective assessments within cognitive aging research. For instance, research based on data from the Ohio Longitudinal Study of Aging and Retirement (OLSAR) has reported similar associations, suggesting that negative self-perception may be linked to cognitive decline through psychological stress or neuroendocrine pathways (29). Consistent with this notion, neuroscience and clinical research have documented that persistent states of anxiety and fear are correlated with alterations in memory-related brain regions such as the hippocampus via chronic activation of the stress response system (30–32). It is noteworthy, however, that the relationship between specific facets of affectivity, such as loneliness, and cognitive decline remains inconsistent across studies. A longitudinal survey of older Chinese adults found that cognitive decline was more strongly and independently associated with social isolation rather than loneliness, with the latter association attenuating after adjusting for depressive symptoms (33). In the present study, however, even after comparable adjustments, the statistical association between the broader PNA construct and cognitive impairment remained robust. One potential explanation for this divergence may lie in our study’s assessment of PNA as a multidimensional construct, which captures a wider spectrum of psychological states beyond the specific experience of loneliness. Multi-item scales, by virtue of encompassing a broader range of emotional dimensions, may not only detect fluctuations with greater sensitivity than single-item measures but also provide a more comprehensive representation of an individual’s enduring emotional landscape, thereby revealing associations that might otherwise be obscured.

Another finding of this study is that, compared with High-stable SLA, Low-stable SLA was associated with higher odds of cognitive impairment. This pattern of association aligns with the perspective that the odds of cognitive impairment among older adults are lower among those with higher levels of social leisure activity. The cognitive reserve hypothesis posits that social leisure engagement may promote synaptogenesis and neurogenesis through the provision of mental or cognitive stimulation. These additional synapses and neurons are thought to exert a compensatory effect within the brain, potentially helping to mitigate the expression of cognitive impairment (34). Consistent with this hypothesis, a cross-sectional study involving 1,214 older adults reported findings suggesting that social opportunities can facilitate multiple communication behaviors essential for interpersonal interactions. These activities have been proposed to positively influence cognitive function via stimulation of neural activity in the brain’s language and executive function regions (35). As an alternative or complementary explanation, the vascular hypothesis suggests that active social engagement and leisure activities may encourage physical activity, which in turn is associated with improved hormone levels and cerebral blood flow. Moreover, vascular risk factors have been strongly linked to the onset and progression of dementia (15, 35). Taken together, these perspectives suggest that SLA may be favorably associated with cognitive functioning in older adults through its potential to promote healthier lifestyles, foster stronger social relationships, and enhance self-regulation (36).

The main contribution of the present study lies in its direct comparison of the magnitude of the statistical associations of PNA and SLA with cognitive impairment, with the observed association being stronger for PNA. Using a group-based trajectory model (GBTM), we identified different developmental patterns in older adults’ affective states and participation in social leisure activities, which revealed two trajectory groups with elevated odds of cognitive impairment: “High-rapid-increasing PNA” and “Low-stable SLA.” To date, few studies have directly compared the magnitude of the associations of PNA and SLA with cognitive decline, and the relative contributions of these two factors remain underexplored. In the present analysis, the association between adverse PNA and cognitive impairment was stronger than that observed for the adverse SLA. One possible framework for interpreting this pattern of results involves a dual-pathway perspective. First, negative affectivity has been proposed to correlate with direct neurological alterations through chronic activation of the stress response system, which has been associated with structural and functional changes in brain regions like the hippocampus (37). Second, PNA may also be indirectly associated with cognitive health through its potential influence on engagement in protective behaviors (38). For instance, reduced motivation related to negative affectivity could diminish older adults’ participation in physical activities, socialization, and sleep hygiene, all of which are thought to support cognitive function. Such a dynamic could contribute to a self-perpetuating cycle wherein negative affectivity is not only statistically associated with poorer cognition but also with reduced engagement in behaviors that might otherwise help maintain cognitive health.

Several strengths of the present study warrant consideration. These include a large and nationally representative sample of Chinese older adults, the application of a novel analytical approach to model longitudinal trajectories, and the robustness of the observed associations. By utilizing data from a 10-year longitudinal cohort, this study examined the dynamic trajectories of PNA and SLA and their respective associations with cognitive function, thereby addressing the limitations inherent in cross-sectional designs. Furthermore, the assessment of PNA utilized a multi-item scale, capturing a broader spectrum of affective dimensions rather than relying on a single indicator, offering a more comprehensive examination of how multifaceted affective states are associated with cognitive function over time.

However, this study has several limitations. First, the temporal direction of the association between negative affectivity and cognitive decline remains unclear. The observed association may reflect a causal influence, stem from reverse causality, or both. Notably, affective symptoms may also represent a prodromal phase or early manifestation of underlying neurodegenerative processes, rather than functioning strictly as an independent antecedent factor. Second, the affectivity measure in this study was a shortened and culturally adapted scale. Although the PNA scale yielded a Cronbach’s α of 0.828 with satisfactory internal consistency, this abbreviated tool lacks formal independent psychometric validation. The full 20-item PANAS was not administered due to the practical constraints of the CLHLS, which may lead to potential construct overlap across affective dimensions. As PNA is a central variable in the present trajectory analysis, such measurement limitations may bias GBTM-based trajectory classification and distort the observed associations between affective profiles and cognitive impairment. In addition, this simplified scale may not fully capture multidimensional affective components, and only reflects subclinical emotional states rather than clinical mood disorders. Third, although the study adjusted for multiple covariates (e.g., age, gender, urban/rural/city residence, education, BMI, smoking and drinking status), unmeasured confounding remains a concern. Several factors known to influence cognitive function, including clinical mood disorders, sleep quality, and vascular comorbidities, were not directly measured or adjusted for in the present analysis. While the consistency of findings across sensitivity analyses supports the robustness of our results, the possibility that these unmeasured factors may have influenced the observed associations cannot be fully excluded. Fourth, prior studies have indicated that the CMMSE has limitations in assessing cognitive function in older adults, including inadequate coverage of certain cognitive domains and the influence of educational attainment and cultural background on its sensitivity (39). Fifth, participant attrition over the decade-long follow-up may have influenced the composition of the analytical sample. While this limitation is inherent to long-term cohort studies, it warrants consideration when interpreting the generalizability of the findings. Sixth, the SLA index was constructed from seven items available in the CLHLS questionnaire. The selected items may not fully represent the spectrum of social leisure activities relevant to Chinese older adults. Consequently, measurement error may attenuate or bias the observed associations.

## 5 Conclusion

The current analysis indicates that older adults characterized by persistently elevated negative affectivity or chronically low social leisure engagement exhibit higher odds of cognitive impairment. Direct comparison of longitudinal trajectories further revealed that while both PNA and SLA were associated with cognitive impairment in older adults, the observed association was substantially stronger for PNA. This pattern derived from a decade of follow-up in a nationally representative cohort provides novel empirical evidence regarding the relative salience of affective versus social leisure engagement factors in the context of cognitive aging. Taken together, these findings contribute to a growing body of research underscoring the relevance of affective dimensions to cognitive health trajectories, and suggest that longitudinal monitoring of emotional well-being may offer incremental value for preserving cognitive health in older adults.

The present findings do call attention to the possibility that affective well-being may represent an underappreciated dimension in cognitive aging research and practice. Should future intervention studies substantiate the directional nature of this association, greater consideration of emotional health in aging policies and community programs may prove warranted.

## Data Availability

Publicly available datasets were analyzed in this study. This data can be found here: https://opendata.pku.edu.cn/dataverse/CHADS.
