# Supplementary material for: Stronger associations of affectivity than social leisure activities with cognitive impairment: a 10-year trajectory study of Chinese older adults
Source: Front Psychiatry. 2026 May 19;17:1842578. doi: 10.3389/fpsyt.2026.1842578 (PMC13226541; doi:10.3389/fpsyt.2026.1842578)
Supplement: Supplementary file 1 [file Supplementaryfile1.docx]

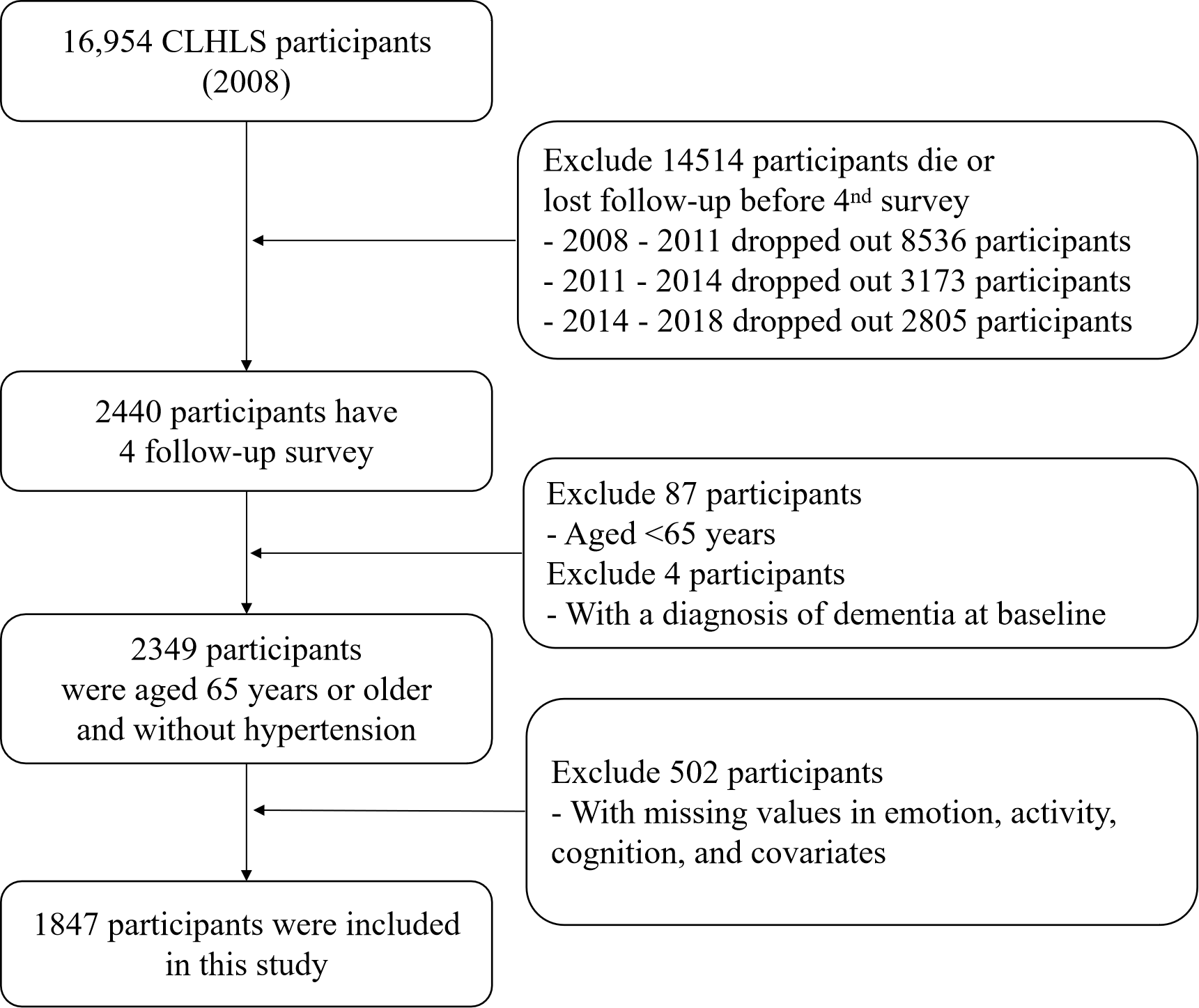


**Figure S1.** Flow chart of the included CLHLS participants

**Table S1.**  List of questionnaire items used to construct the PNA

|  | Characterize | Item | Frequency | PNA |
| --- | --- | --- | --- | --- |
| Positive affectivity | Philosophical optimism | Do you always look on the bright side of things? | always | 1 |
|  |  |  | often | 2 |
|  |  |  | sometimes | 3 |
|  |  |  | seldom | 4 |
|  |  |  | never | 5 |
|  | Autonomy | Can you make your own decisions concerning your personal affairs? | always | 1 |
|  |  |  | often | 2 |
|  |  |  | sometimes | 3 |
|  |  |  | seldom | 4 |
|  |  |  | never | 5 |
|  | Sense of youthfulness | Compared to your actual age, do you usually feel younger, the same, or older? | always | 1 |
|  |  |  | often | 2 |
|  |  |  | sometimes | 3 |
|  |  |  | seldom | 4 |
|  |  |  | never | 5 |
|  | Clean preference | Once you’ve tidied up your surroundings, to what extent do you feel satisfied or at peace? | always | 1 |
|  |  |  | often | 2 |
|  |  |  | sometimes | 3 |
|  |  |  | seldom | 4 |
|  |  |  | never | 5 |
| Negative affectivity | Tension/fear | Do you often feel fearful or anxious? | always | 1 |
|  |  |  | often | 2 |
|  |  |  | sometimes | 3 |
|  |  |  | seldom | 4 |
|  |  |  | never | 5 |
|  | Loneliness | Do you often feel lonely and isolated? | always | 1 |
|  |  |  | often | 2 |
|  |  |  | sometimes | 3 |
|  |  |  | seldom | 4 |
|  |  |  | never | 5 |
|  | Sense of worthlessness | Do you feel the older you get, the more useless you are, and have trouble doing anything? | always | 1 |
|  |  |  | often | 2 |
|  |  |  | sometimes | 3 |
|  |  |  | seldom | 4 |
|  |  |  | never | 5 |

**Table S2.** Model Fitting statistics for PNA trajectories.

| APP | | | |  |  |  |  |  |
| --- | --- | --- | --- | --- | --- | --- | --- | --- |
| Model | G1 | G2 | G3 | AIC | BIC | CAIC | SSBIC | HQIC |
| Traj_1 | 1.000 |  |  | 42762.49 | 42790.13 | 4279.13 | 42777.41 | 42771.99 |
| Traj_2 | 0.993 | 0.997 |  | 41917.45 | 41979.62 | 41988.62 | 41951.02 | 41938.82 |
| Traj_3 | 0.992 | 0.997 |  | 41917.46 | 41979.62 | 41988.62 | 41951.02 | 41938.82 |
| Traj_4 | 0.998 | 0.989 | 0.993 | 41771.13 | 41874.74 | 41889.74 | 41827.08 | 41806.74 |

**Table S3.** Model Fitting statistics for SLA trajectories.

| APP | | | | |  |  |  |  |  |
| --- | --- | --- | --- | --- | --- | --- | --- | --- | --- |
| Model | G1 | G2 | G3 | G4 | AIC | BIC | CAIC | SSBIC | HQIC |
| Traj_1 | 1.000 |  |  |  | 26255.27 | 26289.80 | 26294.80 | 26273.91 | 26267.13 |
| Traj_2 | 0.999 | 0.992 |  |  | 25452.46 | 25528.44 | 25539.44 | 25493.49 | 25478.57 |
| Traj_3 | 0.994 | 0.983 | 0.999 |  | 25449.05 | 25552.67 | 25567.67 | 25585.00 | 25484.66 |
| Traj_4 | 0.987 | 0.993 | 0.988 | 0.999 | 25532.93 | 25684.90 | 25706.90 | 25614.99 | 25585.16 |

**Table S4.** Participants’ characteristics in the follow-up surveys

| Variables | 2011 survey | 2014 survey | 2018 survey |
| --- | --- | --- | --- |
| MMSE score^*^ | 27.01±3.96 | 26.68±4.42 | 24.28±7.48 |
| Cognitive function |  |  |  |
| Normal cognitive function | 1783 (96.53) | 1773 (95.99) | 1585 (85.81) |
| Cognitive impairment | 64 (3.47) | 74 (4.01) | 262 (14.19) |
| Age, M (P25, P75) | 76 (72, 83) | 79 (75, 86) | 83 (79, 90) |
| Residency, n (%) |  |  |  |
| City | 294 (15.92) | 300 (16.24) | 339 (18.35) |
| Urban | 625 (33.84) | 665 (36.00) | 715 (38.71) |
| Rural | 928 (50.24) | 882 (47.75) | 793 (42.93) |
| BMI, n (%) |  |  |  |
| Normal weight | 1098 (59.45) | 1169 (63.29) | 1069 (59.34) |
| Underweight | 554 (29.99) | 485 (26.26) | 534 (28.91) |
| Overweight | 56 (3.03) | 61 (3.30) | 67 (3.63) |
| Obesity | 139 (7.53) | 132 (7.15) | 150 (8.12) |
| Smoking status |  |  |  |
| Never smoker | 1175 (63.62) | 1227 (66.43) | 1220 (66.05) |
| Former smoker | 373 (20.19) | 349 (18.90) | 303 (16.40) |
| Current smoker | 299 (16.19) | 271 (14.67) | 324 (17.54) |
| Alcohol consumption |  |  |  |
| Never drinker | 1193 (64.59) | 1299 (70.33) | 1316 (71.25) |
| Former drinker | 261 (14.13) | 213 (11.53) | 248 (13.43) |
| Current drinker | 293 (21.28) | 335 (18.14) | 283 (15.32) |

### **Table S5.** Longitudinal changes in MMSE scores from 2008 to 2018, stratified by trajectory groups

| Trajectory Group | n | MMSE 2008  （Mean±SD） | MMSE 2018  （Mean±SD） | Change  (95% CI) |
| --- | --- | --- | --- | --- |
| **PNA Trajectories** |  |  |  |  |
| Low-stable-increasing PNA | 1398 | 27.5±3.3 | 26.0±5.0 | -1.5 (-1.8，-1.2) |
| High-rapid-increasing PNA | 449 | 24.9±6.8 | 18.8±10.7 | -6.0 (-7.1，-5.0) |
| **SLA Trajectories** |  |  |  |  |
| Low-stable SLA | 610 | 25.3±5.8 | 21.2±9.1 | -4.0 (-4.8，-3.3) |
| High-stable SLA | 1237 | 27.7±3.5 | 25.8±5.9 | -1.9 (-2.3，-1.6) |

**Table S6.** The associations between PNA, SLA and cognitive function in 2008

|  | Variables | Normal cognitive function | Cognitive impairment |
| --- | --- | --- | --- |
| PNA crude model  OR (95% CI) | High-rapid-increasing PNA  (Ref. Low-stable-increasing PNA) | Reference | 5.12 (3.17, 8.30) |
| PNA adjusted model  OR (95% CI) | High-rapid-increasing PNA  (Ref. Low-stable-increasing PNA) | Reference | 2.78 (1.66, 4.66) |
| SLA crude model  OR (95% CI) | Low-stable SLA  (Ref. High-stable SLA) | Reference | 4.14 (2.53, 6.78) |
| SLA adjusted model  OR (95% CI) | Low-stable SLA  (Ref. High-stable SLA) | Reference | 2.10 (1.22, 3.62) |
| PNA and SLA simultaneous model  OR (95% CI) | High-rapid-increasing PNA  (Ref. Low-stable-increasing PNA) | Reference | 2.45 (1.44, 4.19) |
|  | Low-stable SLA  (Ref. High-stable SLA) | Reference | 1.67 (0.94, 2.94) |

Note: OR=Odds ratio

Adjusted models were adjusted for age (continous), sex (male or female), residency (city/urban/rural), illiteracy (yes/no) , BMI (normal weight, underweight, overweight or obesity), smoking and drinking status (never/ former/current smokers/drinkers).

PNA: Positive and Negative Affectivity; SLA: Social Leisure Activities

**Table S7.** The associations between PNA, SLA and cognitive function, using education-specific cut-off points.

|  | Variables | Normal cognitive function | Cognitive impairment |
| --- | --- | --- | --- |
| PNA crude model  OR (95% CI) | High-rapid-increasing PNA  (Ref. Low-stable-increasing PNA) | Reference | 5.22 (4.37, 6.25) |
| PNA adjusted model  OR (95% CI) | High-rapid-increasing PNA  (Ref. Low-stable-increasing PNA) | Reference | 3.58 (2.95, 4.35) |
| SLA crude model  OR (95% CI) | Low-stable SLA  (Ref. High-stable SLA) | Reference | 3.45 (2.88, 4.12) |
| PNA adjusted model  OR (95% CI) | Low-stable SLA  (Ref. High-stable SLA) | Reference | 2.28 (1.86, 2.78) |
| PNA and SLA simultaneous model  β (95% CI) | High-rapid-increasing PNA  (Ref. Low-stable-increasing PNA) | Reference | 3.16 (2.58, 3.86) |
|  | Low-stable SLA  (Ref. High-stable SLA) | Reference | 1.72 (1.40, 2.12) |

Note: OR=Odds ratio

Adjusted models were adjusted for age (continous), sex (male or female), residency (city/urban/rural), illiteracy (yes/no) , BMI (normal weight, underweight, overweight or obesity), smoking and drinking status (never/ former/current smokers/drinkers).

PNA: Positive and Negative Affectivity; SLA: Social Leisure Activities
